# Participation in a food assistance program and excessive weight gain: an evaluation of the Brazilian Worker’s Food Program in male and female manufacturing workers

**DOI:** 10.1186/s12889-022-13447-8

**Published:** 2022-06-04

**Authors:** Raiane Medeiros Costa, Ingrid Wilza Leal Bezerra, Karina Gomes Torres, Gabriela Santana Pereira, Anissa Melo de Souza, Antonio Gouveia Oliveira

**Affiliations:** 1 Postgraduate Program in Health Sciences, Centro de Ciências da Saúde, Universidade Federal do Rio Grande do Norte, Av. Nilo Peçanha 620, Petrópolis, Natal, RN 59012-300 Brazil; 2 Nutrition Department, Centro de Ciências da Saúde, Universidade Federal do Rio Grande do Norte, Av. Senador Salgado Filho, s/n, Lagoa Nova, Natal, RN 59078-970 Brazil; 3 Pharmacy Department, Centro de Ciências da Saúde, Universidade Federal do Rio Grande do Norte, Av. Nilo Peçanha 620, Petrópolis, Natal, RN 59012-300 Brazil

**Keywords:** Public policy, Body mass index, Food assistance programs, Diet, food and nutrition, Occupational health

## Abstract

**Background:**

Several published studies have reported an association between participation in a food assistance program and greater prevalence of overweight/obesity. Our aim was to compare nutritional status and nutrient consumption between workers from manufacturing companies participant and non-participant in the Brazilian Workers’ Food Program (WFP).

**Design:**

Cross-sectional survey, based on a probability sample of manufacturing workers in Brazil obtained by stratified two-stage sampling, comparative between WFP and non-WFP participating companies. Body mass index (BMI), waist circumference (WC), and nutrient consumption (24-hour recall) were collected by trained nutritionists. Statistical analysis was done separately in each sex with mixed effects multilevel linear regression model including sampling weights and covariate adjustment.

**Results:**

Thirty-three companies were randomly selected from all companies in three different economic activity sectors (food and beverages, non-metallic minerals, and textiles) in North-eastern Brazil, with stratification by company size, and a random sample of 929 workers (484 from non-WFP and 445 from WFP companies) was obtained from those companies. In males, the WFP group had higher BMI (+ 1.08 kg/m^2^, *p* < 0.001), greater WC (+ 3.27 cm, *p* < 0.001) and greater prevalence of obesity (OR 1.67, *p* < 0.001). In females, no statistical significant differences were observed in anthropometric parameters, but the WFP group had lower prevalence of obesity (OR 0.49, *p* = 0.05). Among workers in companies that provide lunch, males from WFP companies have greater consumption of carbohydrates (+ 39.5 kcal, *p* = 0.02) and protein (+ 11.1 kcal, *p* = 0.08), while females have lower protein consumption (− 14.2 kcal, *p* = 0.04) and also lower total daily consumption of carbohydrates (− 59.3 Kcal, *p* = 0.05) and total lipids (− 14.2 Kcal, *p* = 0.04).

**Conclusions:**

Participation in the WFP is associated with increased BMI and WC among male workers; however, this association was not found in females. Compared to the non-WFP group, in the WFP group, males have greater consumption of carbohydrates and protein at lunch, while women have lower protein intake. These results indicate the need that proposals for public policies aimed to the improvement of the nutritional status of populations take into consideration the different effects of food assistance programs in males and females.

**Supplementary Information:**

The online version contains supplementary material available at 10.1186/s12889-022-13447-8.

## Background

The issue of food and nutrition has mobilized organizations and countries to create and implement public policies aimed at guaranteeing and enforcing the Human Right to Adequate Food (HRAF), with emphasis on Food and Nutrition Security (FNS) actions in its several dimensions: availability, access, consumption, production and biological use of food [1, 2]. In Brazil, the Federal Constitution warrants the HRAF, through which the Brazilian State has the obligations to respect, protect, promote and provide food for the population while, in turn, the population has the right to demand that their rights be assured through enforceability mechanisms [3]. The National Food and Nutrition Policy (NFNP) is the main public policy aimed at securing the HRAF for the general population, with specific programs and actions aimed at different population groups such as, among others, schoolchildren, childbearing women, the elderly, and workers. Regarding the latter group, the Workers’ Food Program (WFP), a government policy set forth in 1976 [4], whose regulation was revised in 2021 [5], has as main objective to improve the nutritional status of workers, mainly in the low-income groups, through the provision of nutritionally adequate and healthy food, either as meals offered in refectories within the workplace which almost always are available to all the workers, as food baskets, or as vouchers for purchasing ready meals in restaurants or for purchasing food at the supermarket, as long as both are accredited to the WFP and meet the Program’s nutritional parameters, with a worker’s co-payment of no more than 20% of the meal costs. Participation in the WFP is open to all companies that comply with certain fiscal requirements and, in turn, they have benefit of a number of tax exemptions, including a tax deduction of 4% of the income tax due. When the company chooses to offer meals in its own refectory, its workers now have daily access to at least one main meal - lunch, dinner or supper, depending on their working hours or shift [6, 7]. The importance of the WFP is reflected in its numbers: according to Government data, as of 2021 the program covered over 22 million workers from nearly 287 thousand participating companies, representing a value of 183 million USD in tax exemption [8, 9].

The role of the WFP is to contribute food security, thereby fulfilling the right to regular and permanent access to an adequate and healthy diet, as well as carrying out the nutritional diagnosis of workers, as established by the Ordinance that determines the WFP’s nutritional parameters [10].. The WFP is supervised by a Tripartite Commission, formed by representatives of the government, stakeholders and workers, who are responsible for evaluating and monitoring its execution, through review of information collected yearly from each adherent company, as well as imposing sanctions in case of non-compliance with its rules, which may include exclusion from the program and loss of the tax benefits [11].

Although it is naturally expected that a food assistance program will improve the nutritional status of their participants, there is empirical evidence that the WFP has fallen short to achieving its objectives. Actually, several studies have revealed that the meals served to workers, whether by the company or by outsourced companies, are often nutritionally inadequate when compared to the legal requirements [12–14]. Briefly, these define that the main meals must contain 30-40% of the daily total energy with the following distribution of nutrients: carbohydrate 60%, protein 15%, total fat 25%, saturated fats: < 10, fibre 7-10 g, and sodium 720-960 mg [10]. In addition, other studies have found a positive association between participation in the WFP and increased weight gain [15, 16], higher prevalence of overweight and obesity [17] and greater obesity-related cardiovascular risk [18]. These do not seem to be isolated findings related to the WFP, since similar results have been described in evaluations of food assistance programs for low-income people in the United States as well as in other countries around the world [19–22].

However, with rare exceptions, those studies have been based on secondary data sources or, when based on direct evaluations, were not comparative and generally based on convenience samples. Therefore, in order to contribute to the clarification of whether the WFP has the unintentional effect of increasing weight, we conducted a population survey with direct interview of workers from manufacturing industries with the objective of investigating an association between participation in a food assistance program and weight gain, by direct comparison of body mass index, waist circumference and nutrients consumption of male and female workers between companies adherent and non-adherent to the WFP.

## Subjects and methods

From September 2017 to April 2018 we conducted an observational cross-sectional study, comparative between workers from manufacturing industries adherent and non-adherent to the WFP. The study took place in the State of Rio Grande do Norte, with a population of 2.8 million and located in northeaster Brazil.

The study was limited to the sectors of activity most important to the State’s economy: food and beverages, non-metallic minerals, and textiles. The survey was done separately in WFP-adherent and non-adherent companies and was based on a combined stratified and two-stage sampling plan. For WFP companies, the offer of meals (lunch or dinner) to workers was used as inclusion criteria. The stratification factors were the sector of activity, and the size of the company categorized as small (20 to 99 workers), medium (100 to 499) and large (500 or more). The first stage consisted of companies selected by simple randomization, in proportion to the number of companies active in the State in each stratum. The randomization was done with computer-generated random numbers from the list of all companies active in the State maintained by the Federation of Industries of the State of Rio Grande do Norte (FIERN). After the selected companies agreed in writing to participate in the research, a professional from the companies’ human resources department was contacted to facilitate locally the set-up of the survey. This individual informed the workers of the dates of the scheduled visits, which were randomized in each company. In order to avoid interference with the factories’ production schedule, data collections were performed from Tuesday to Saturday, at a time defined in accordance with the convenience of the company, in the morning shift before the worker received the meal or immediately before the afternoon shift. One to four visits were made to each company, depending on the size of the company. Each company provided a space (cafeteria, auditorium, ambulatory) equipped with the necessary material to conduct the interviews.

Another function of the company’s representative was to provide a roster with the identification of the workers who frequented the cafeteria at lunchtime that verified the inclusion and exclusion criteria. The inclusion criteria were age over 18 years-old and employed for over 1 year in the company. Trainees and other temporary workers, as well as pregnant women were excluded. The workers eligible for the survey were selected from this list by simple randomization, also with computer-generated random numbers, and were enrolled into the study after signing the informed consent form. No randomized worker declined participation in the study and there were no consent withdrawals.

Collected data included bio-demographic data (age, sex, education, marital status, monthly income, participation in in-house professional training actions), weight using an Inner Scan digital scale (Tanita Corp., Tokyo, with an accuracy of ±0.05 kg), height using portable stadiometers (Sanny, São Bernardo do Campo, SP, Brazil), waist circumference (WC) measured at the midpoint between the lower edge of the last rib and the iliac crest.

The worker’s physical activity level was valuated with a validated Portuguese translation of the short version of the International Physical Activity Questionnaire (IPAQ), which estimates the Metabolic Equivalent of Task per minute per week (MET.min/week) from self-reported amount of time spent in the previous 7 days in vigorous physical activity, moderate physical activity, walking and sitting [23, 24].

The 24-hour recall method was used for the collection of information on nutrient consumption in all the meals from the prior day, specifying the time and place of the meal, in addition to the details of the preparation of meals including reported home measures. All the information collected was anonymized in order to protect the workers’ privacy.

All study procedures were performed by nutritionists and graduate students from the nutrition course at the Federal University of Rio Grande do Norte (UFRN), who had been previously trained according to the techniques recommended in the guidelines for the collection and analysis of anthropometric data from the SISVAN (Food Surveillance System and Nutrition) - Ministry of Health [25].

### Statistical analysis

Formal methods for the computation of sample size have not yet been developed and an often adopted recommendation is that 30 clusters with a cluster size of 30 are probably adequate to obtain unbiased estimates of effect sizes in complex survey plans analysed with multilevel models [26]. Accordingly, the target sample size was defined at 900 workers from 30 companies.

Body mass index (BMI) was computed as body weight in kilograms divided by the square of body height in meters. The data from the 24-hour dietary recall were analysed using the Brazilian Table of Food Composition TACO [27], complemented whenever necessary by other composition tables, [28–30], as well as from the nutrition facts labels. For classification of cardiometabolic risk according to WC, the following WC cut-offs were used: increased risk ≥94 cm for men and ≥ 80 cm for women; substantially increased risk ≥102 cm for men and ≥ 88 cm for women [31].

Statistical analysis was performed for each sex separately. Population estimates of bio-demographic characteristics in each sex and group were computed according to the combined stratified and multistage survey design, including base survey weights, and finite population correction for companies using information provided by FIERN.

The bio-demographic variables were compared between the groups for the identification of potential confounding factors, with mixed effects multilevel logistic regression using survey weights equal to the reciprocal of the probability of a company being selected for the survey, and of the probability of a worker being selected conditional to the probability of their company having been selected. The model specification included stratification by company size, with group (WFP and non-WFP) as fixed factor, and with sector of activity, company nested within sector of activity and workers nested within companies as random factors. Because of the non-normal distribution of those variables, with the exception of age, they were dichotomized at their median value. Physical exercise was dichotomized at 700 MET.min/week, a cut-off corresponding to the recommended weekly exercise activity according to the 2019 ACC/AHA guidelines18 (approximately 150 minutes per week of moderate-intensity, or 75 minutes per week of vigorous-intensity, physical exercise) [32].

For the between groups comparison of the main study variables BMI, WC, and nutrient consumption, mixed effects multilevel linear regression was used with the same stratification and set of fixed and random factors, but also including covariates identified in the above analysis. For the analysis of differences between sexes of the relationship of the WFP with the study variables, we tested the interaction of sex with WFP using the same model described above. Mixed effects multilevel logistic regression with the same model specifications as above was used for the between groups comparison of the prevalence of increased cardiometabolic risk, with testing of WFP by sex interaction to detect differences between sexes. In all analysis, an independent covariance structure and robust standard errors with the sandwich estimator were used. Two-tailed *p*-values < 0.05 were considered indicative of statistically significance. Stata 15 (Stata Corporation, College Station, TX, USA) was used in all analyses. The study data is available from the Supplementary Material.

## Results

A total of 929 workers in 33 companies were evaluated, with 484 workers from non-WFP companies and 445 workers from WFP companies. Table 1 shows the distribution of companies and study participants in each group and each stratum.

**Table 1:** Description of study strata and sample sizes in the two-stage survey design

Characteristics of the surveyed industries	Workers’ Food Program		non-Workers’ Food Program	
	Number of companies	Number of workers	Number of companies	Number of Workers
Sector of activity				
Food and beverages	7	189	7	200
Non-metallic minerals	3	86	3	93
Textile	6	170	7	191
Company size				
Small	6	133	7	168
Medium	7	177	7	168
Large	3	135	3	148

Table 2 shows the population estimates of the bio-demographic and socio-economic characteristics of the workers in the two study groups (WFP and non-WFP) in each sex.

**Table 2:** Comparison of characteristics of workers between industries adherent and non-adherent to the Brazilian WFP

Variable	Male workers		***p***	Female workers		***p***
	WFP	non-WFP		WFP	non-WFP	
	Estimate (95% CI)	Estimate (95% CI)		Estimate (95% CI)	Estimate (95% CI)	
Age, years (mean)	38.7 (36.5; 41.0)	34,1 (32.2; 36.1)	0.06	39.9 (38.0; 41.8)	41.9 (40.0; 43.8)	0.68
Married (%)	69.3 (59.5; 77.6)	61,1 (51.3; 70.0)	**0.007**	59.6 (48.4; 69.8)	55.2 (45.3; 64.7)	0.97
Children (%)	71.8 (62.2; 79.7)	58.4 (49.2; 67.2)	0.36	72.0 (60.0; 81.5)	78.9 (69.6; 85.9)	0,76
Education^a^ (%)	65.8 (56.4; 74.1)	55.0 (46.0; 63.7)	0.37	73.7 (60.8; 83.5)	52.1 (40.4; 63.6)	< 0.001
Income^b^ (%)	64,1 (54.5; 72.7)	64.9 (54.0; 74.5)	0.91	30.1 (21.3; 40.7)	17.5 (11.0; 26.7)	0.10
In-house formation (%)	31.3 (22.9; 41.2)	17.8 (11.4; 26.8)	0.85	17.4 (10.4; 27.6)	10.9 (5.7; 19.8)	0.41
Physical exercise^c^ (%)	67.0 (57.7; 75.1)	44.7 (36.2; 53.4)	0.39	93.6 (88.5; 96.5)	88.8 (81.0; 93.7)	0.81

Mixed effects multilevel logistic regression stratified by company size (fixed factors: WFP; random factors: activity sector, company nested within sector and worker nested within company). ^a^High school or higher education; ^b^Monthly income above one minimum wage (954 BRL or about 170 USD); ^c^Above 700 METS.min/week

The only statistically significant differences between workers of WFP and non-WFP companies was a greater frequency of married male workers in WFP companies (69.3% vs. 61.1%), *p* = 0.007) and greater educational level of female workers in WFP companies (73.7 vs. 52.1% with high school or greater education, *p* < 0.001). There were statistically significant differences between sexes in those variables: male workers were younger (− 1.9 years, *p* = 0.002), more often married (odds ratio (OR) 1.57, *p* < 0.001) and without children (OR 0.77, *p* = 0.08), had higher wages (OR of income above 1 minimum wage 3.58, *p* = 0.01) and less likely to achieve the recommended amount of physical exercise (OR 0.17 of more than 700 MET.min/week 0.17, *p* < 0.001). All analyses involving between sexes comparisons were adjusted by those variables.

In the comparison of the anthropometric indicators of nutritional status between workers from WFP and non-WFP companies (Table 3), male workers from WFP companies had a greater mean BMI, on average 1.08 kg/m2 greater than non-WFP workers (*p* < 0.001). In female workers the higher mean BMI was observed among workers of non-WFP companies, but the difference was not statistically significant (*p* = 0.17). The test of the interaction of WFP with sex was significant (*p* = 0.007), supporting the conclusion that the relationship between WFP and BMI is different in the two sexes. Regarding WC, the mean value was also higher in male workers from WFP companies, on average 3.27 cm (*p* < 0.001), while female workers from non-WFP companies had larger estimated WC, but the difference was not statistically significant (*p* = 0.17). Again, the relationship between WFP and WC was also different between sexes (p for interaction =0.001).

**Table 3:** BMI and WC in workers from industries adherent and non-adherent to the Brazilian WFP

Variable	WFP	non-WFP	Difference	***p***	Interaction
	Mean (95% CI)	Mean (95% CI)	Mean (95% CI)		***p***
Body Mass Index (kg/m2)					
Male^a^	27.4 (26.5; 28.3)	25.9 (25.2; 26.7)	1.08 (0.73; 1.44)	< 0.001	0.007
Female ^b^	27.0 (26.1; 28.0)	29.0 (27.9; 30.1)	−1.61 (−3.90; 6.77)	0.17	
Waist circumference (cm)					
Male ^a^	92.8 (90.4; 91,0)	89.0 (86.9; 91.0)	3.27 (1.83; 4.70)	< 0.001	0.001
Female ^b^	87.2 (84.9; 89.4)	91.3 (88.6; 93.9)	−3.66 (−8.86; 1.55)	0.17	

Mixed effects multilevel linear regression stratified by company size (fixed factors: WFP; random factors: activity sector, company nested within sector and worker nested within company)

^a^: analysis adjusted by marital status; ^b^: analysis adjusted by education level

**Fig. 1:**
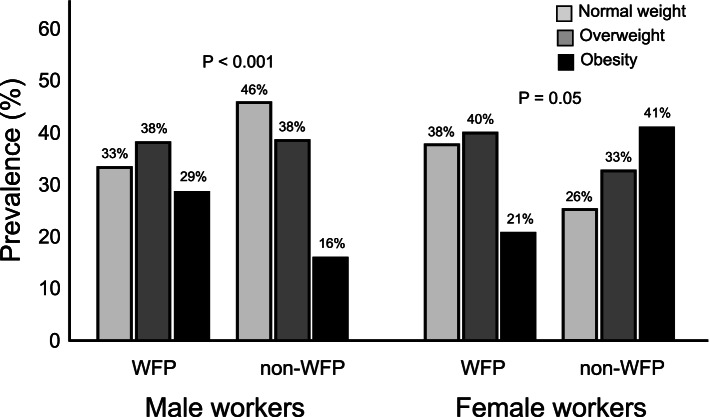
Nutritional status of workers from industries adherent and non-adherent to the Brazilian WFP by sex. Legend: *P*-values are from mixed effects multilevel logistic regression stratified by company size (fixed factors: WFP; random factors: activity sector, company nested within sector and worker nested within company) with adjustment by marital status in males and education level in females

When comparing the WFP and non-WFP groups by class of nutritional status as a function of BMI values, the relationship between the WFP and class of nutritional status was different between sexes (p for interaction < 0.001) (Fig. 1), with a higher prevalence of obesity observed among men in the WFP group (OR 1.67, 95% confidence interval (CI) 1.31 – 2.14, *p* < 0.001), while the reverse was observed among women (OR 0.49, 95% CI 0.24 – 1.00, *p* = 0.05).

**Fig. 2:**
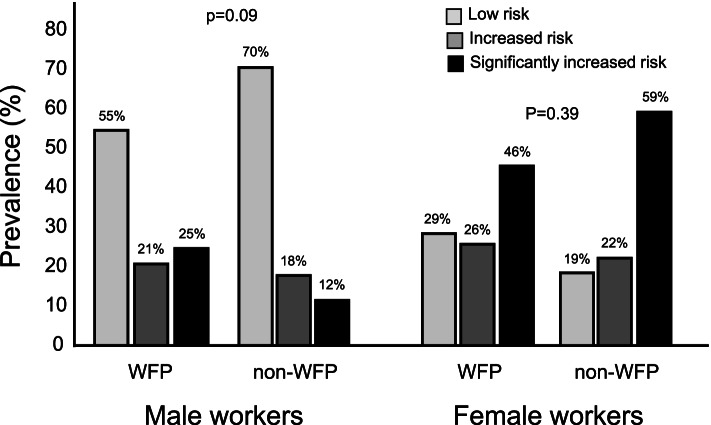
Cardiometabolic risk of workers from industries adherent and non-adherent to the Brazilian WFP by sex. Legend: P-values are from mixed effects multilevel logistic regression stratified by company size (fixed factors: WFP; random factors: activity sector, company nested within sector and worker nested within company) with adjustment by marital status in males and education level in females

In the comparison of the distribution of cardiometabolic risk classification according to WC (Fig. 2), men in the WFP group had a higher frequency of increased cardiometabolic risk (OR 2.04, 95% CI 0.88 – 4.73, *p* = 0.09), while among women there was no evidence of increased cardiometabolic risk (OR 0.61, 95% CI 0.20 – 1.88, *p* = 0.39), with significant WFP by sex interaction (*p* < 0.001).

Table 4 describes the comparison of energy and nutrient consumption between workers in the two groups, separately for each sex.

**Table 4:** Calorie and nutrient consumption in workers from industries adherent and non-adherent to the Brazilian WFP

Variable	Male workers^a^		Female workers^b^		Interaction Sex × WFP
	Difference WFP to non-WFP (95% CI)	***p***	Difference WFP to non-WFP (95% CI)	***p***	***p***
Energy lunch (Kcal)	36.5 (−75.4; 148.3)	0.52	−59.1 (−141.6; 23.4)	0.16	0.09
Energy other meals (Kcal)	−35.5 (−117.1; 46.0)	0.39	−33.9 (−100.5; 32.7)	0.95	0.52
Energy daily total (Kcal)	3.7 (− 180.8; 188.3)	0.97	−92.5 (−154.3; −30.8)	0.003	0.74
Protein lunch (Kcal)	11.1 (−1.46; 23.6)	0.08	−14.2 (−27.6; −0.7)	0.04	0.69
Protein other meals (Kcal)	3.17 (−10.0; 16.3)	0.64	−0.3 (−25.1; 24.4)	0.98	0.10
Protein daily total (Kcal)	16.6 (−5.28; 38.4)	0.14	−15.5 (− 51.8; 20.9)	0.41	0.32
Lipid lunch (Kcal)	−12.6 (−81.3; 56.2)	0.72	−3.0 (−43.2; 37.3)	0.89	0.66
Lipid other meals (Kcal)	−7.8 (−78.2; 62.7)	0.83	−14.6 (−47.9; 18.6)	0.39	0.17
Lipid daily total (Kcal)	−17.2 (− 148.8; 114.3)	0.78	− 18.6 (−36.0; − 1.3)	0.04	0.81
Carbs lunch (Kcal)	39.5 (6.10; 72.9)	0.02	−43.5 (−101.7; 14.6)	0.14	< 0.001
Carbs other meals (Kcal)	−30.5 (− 40.4; −20.7)	< 0.001	− 17.5 (− 30.1; − 4.9)	0.007	< 0.001
Carbs daily total (Kcal)	6.4 (− 40.4; 53.2)	0.79	−59.3 (− 117.9; −0.8)	0.05	0.07
Saturated fat lunch (Kcal)	− 1.0 (−9.6; 7.6)	0.82	−1.2 (− 14.9; 12.4)	0.86	0.08
Saturated fat other meals (Kcal)	0.9 (− 31.5; 33.3)	0.96	−8.7 (− 23.5; 6.1)	0.25	0.51
Saturated fat daily total (Kcal)	0.2 (−40.7; 41.2)	0.99	−11.1 (−29.2; 7.0)	0.23	0.34
Fiber lunch (g)	1.4 (1.0; 1.8)	< 0.001	−1.4 (−5.2; 2.4)	0.47	0.13
Fiber other meals (g)	−1.2 (− 1.8; −0.6)	< 0.001	0.1 (− 0.9; 1.1)	0.82	0.43
Fiber daily total (g)	0.1 (−0.2; 0.4)	0.43	−1.6 (−6.2; 3.0)	0.50	0.42
Sodium lunch (mg)	−75.4 (− 346.1; 195.3)	0.59	−169.1 (− 244.0; −94.1)	< 0.001	0.55
Sodium other meals (mg)	−46.8 (− 146.1; 52.5)	0.36	−328.8 (− 647.5; − 10.2)	0.04	0.80
Sodium daily total (mg)	− 112.4 (− 306.8; 82.1)	0.26	−500.0 (− 794.2; − 205.7)	0.001	0.98

Mixed effects multilevel linear regression stratified by company size (fixed factors: WFP; random factors: activity sector, company nested within sector and worker nested within company)

^a^ analysis adjusted by marital status; ^b^ analysis adjusted by education level

Men in the WFP group have greater consumption at lunch of carbohydrate (more 39.5 Kcal on average, *p* = 0.02) and lower consumption in the remaining meals (less 30.5 Kcal, p < 0.001); males also had increased consumption of protein at lunch (more 11.1 Kcal in average) which was close to statistical significance (*p* = 0.08). In contrast, women in the WFP group have lower consumption of carbohydrate throughout the day, with statistically significant differences from non-WFP female workers at other meals (less 17.5 Kcal, *p* = 0.007) and daily total (less 59.3 Kcal, *p* = 0.05), as well as lower consumption of protein at lunch (less 14.2 Kcal, *p* = 0.04), and daily total lipids (less 18.6 Kcal, *p* = 0.04). Women in the WFP group also have lower intake of sodium at lunch (less 169 mg, *p* < 0.001), other meals (less 329 mg, *p* = 0.04) and daily total (less 500 mg, *p* = 0.001). Male workers in the WFP group have greater consumption of fibre at lunch (more 1.4 g in average, *p* < 0.001) and lower consumption in the other meals (less 1.2 g, *p* < 0.001). In the analysis of differences between the sexes in the association of the WFP with the consumption of nutrients it was found that, compared to men, in women the differences in nutrient consumption between WFP and non-WFP groups are in the opposite direction regarding energy at lunch (interaction *p* < 0.10), and carbohydrates at lunch and daily total (interaction *p* < 0.05) and in the same direction but in carbohydrates in the other meals (*p* < 0.05).

## Discussion

This cross-sectional, stratified and two-stage survey of a population of manufacturing workers has shown that participation in a food assistance program is associated with increased BMI and WC in males but not in women, the latter presenting lower prevalence of obesity. In addition, male workers participating in the program have higher consumption of carbohydrates and protein in the company provided meals, while women show lower daily consumption of carbohydrates and total lipids, and protein at lunch.

Workplace wellness programs can not only improve health, but also reduce labour costs and increase productivity. In Brazil, the WFP is part of a set of actions that currently comprise the National Food and Nutrition Policy, which addresses the issues of food and nutrition security, but in this case aimed specifically at workers in the formal labour market. However, like all political action, implementation must be accompanied by systematic surveillance and assessments in order to analyse progress and guide further efforts [33]. In the case of the WFP, which is the main food security policy for Brazilian workers, it is relevant to conduct comparative studies that evaluate its impact on workers’ health. Therefore, it is necessary to examine the effects of long-term policies in different locations and between different populations, as well as the best implementation practices, to increase the evidence base in that field [34].

In agreement with that line of thought, the present study assessed the body weight and waist circumference of a probability sample of workers in WFP-adherent industries, using as controls workers from industries in the same geographical region, in the same activity sectors, and from companies of equivalent sizes. The results of this study showed that male workers in the WFP group, compared to men from the non-WFP group, presented greater BMI and WC in average, as well as higher prevalence of obesity and greater cardiometabolic risk. The association of WFP with BMI and WC was not found in female workers, but they did have lower prevalence of obesity. The statistically significant WFP by sex interactions indicate that the relationships of WFP participation with BMI and WC are different between sexes.

The International labour Organization (ILO) develops programs for the Promotion of Workers’ Health and Well-being at Work, with the aim of promoting adequate protection for the life and health of workers. In addition, the World Health Organization (WHO) and the ILO share the understanding of occupational health as responsible for “promoting and maintaining the highest degree of physical, mental and social well-being of workers in all occupations”, recognizing nutrition as part of a healthy work environment [35].

Worldwide, countries like the United Kingdom, Hungary, Romania, France and Sweden, in response to a social need, adopt voucher programs to provide food for workers or offer donations to companies to promote health in the workplace, as is the example of Singapore [35]. Another initiative carried out through a public-private partnership is the European Program FOOD (Fighting Obesity through Offer and Demand), an important program that aims to promote a healthy lifestyle and balanced diet in the workplace, improving the nutritional quality of foods offered in restaurants, to facilitate consumer choice. Its line of action consists of disseminating information and guidance on healthy eating, as well as providing training for employers and restaurant employees [36, 37]. In Brazil, the main public food safety policy aimed at workers is the WFP [16–18, 38]. In addition to these, the FEAD programs in Europe [22] and the SNAP in the United States [39–42] stand out, which are not specific for workers, but whose coverage also serves the working-age population that is in a situation of food and nutritional vulnerability. Some common results have pointed to an association of food assistance programs as FEAD and SNAP with higher BMI, higher WC, and increased prevalence of overweight/obesity, with different effects on men and women, in line with our results.

In Europe, the Fund for European Aid to the Most Deprived (FEAD) was created in 2014 replacing the European Union food distribution program for the most deprived people (MDP) in the community (1987-2013) [43] to help alleviate the worst forms of poverty in the European Union (EU) and to promote the social inclusion of the most deprived people. It provided 3.8 billion EUR of EU funding for the 2014-2020 programming period 4.5 billion EUR including co-financing by Member States), and on average supported almost 13 million people per year during the 2014-2018 period. Member States cooperate with partner organizations (public or non-profit organizations) through two types of operational programs that complement national programs. One of those programs focuses on food assistance, providing meals, food and basic consumption goods. In addition, it is complemented by accompanying measures to promote the social inclusion of final beneficiaries, for example, referring them to appropriate services, and offering guidance on a balanced diet. Five countries have clearly higher allocations of FEAD funding, namely Italy, Spain, France, Poland and Romania. Greece and Portugal follow [44, 45].

In the United States, the Supplemental Nutritional Assistance Program (SNAP), formerly known as the Food Stamp Program (FSP), is considered the largest food assistance program in the country. Although being a federal government program, it is run by State and local agencies and consists of an aid to help low-income families pay for food. To participate, individuals must have a gross monthly income ≤130% of the federal poverty level [46, 47]. There are some differences between the SNAP and the WFP. In SNAP, program participation rests with the individual’s initiative, while in WFP participation is not an individual choice. On the other hand, the WFP provides regular and constant food supply to the program recipients, while the SNAP consists of a single lump sum given at the beginning of each month [48] and the majority of SNAP recipients tend to overeat in the first weeks of the month and reduce consumption later in the month when the funds become scarce [49].

With regard to WFP, consistent with our results, several studies carried out in Brazil have also shown a relationship between weight gain and participation in the program. Two retrospective cohort studies based on the database of the Industry Social Service in the state of Bahia, also found a positive association between participation in food assistance policies and increased weight gain [15, 16]. In a cross-sectional study conducted on 1339 workers from WFP-adherent companies in the State of São Paulo, a high prevalence of overweight and obesity was observed [17]. Two other studies based on probability samples of industries and workers in the State of Rio Grande do Norte, comparing workers between WFP to non-WFP industries, one a cross-sectional [18] and the other a cohort study [38], also found higher prevalence of overweight and obesity, as well as increased cardiovascular risk among workers in WFP industries. However, none of these studies evaluated the association on this population separately in male and female workers.

The results of our study, showing that a food assistance program in low-income people are associated with increased rates of overweight and obesity in males while such associations were not observed among females, has been shown previously in the findings of a survey conducted in 72 municipalities in Greece among 499 recipients of the FEAD program and 500 age-matched controls. Using self-reported anthropometric information and nutritional intake assessed by the food frequency questionnaire, the authors concluded that FEAD participants had a higher prevalence of overweight and obesity compared to controls (overweight 44.0% vs. 37.5%, and obesity 25.4% vs. 18.0%, in FEAD vs Control, respectively), with women in the FEAD group having lower obesity rates than controls (39.0% vs. 48.1%) [22].

In the U.S., several studies have evaluated the nutritional status of low-income people covered by SNAP. A study by Chaparro et al. (2017) [20] using data from the Los Angeles Family and Neighbourhood Survey (LAFANS-2000-2002) on 1176 adults, found that compared to eligible non-participants, SNAP participants had twice the prevalence of obesity (30 vs. 16%), but sex differences were not investigated. In another study on approximately 8000 people eligible to the SNAP and other food assistance programs, using data from the California Health Interview Survey (CHIS) collected by telephone, Leung and Villamor (2011) [21] found that among SNAP male participants the BMI was in average 2.5 kg/m^2^ greater (*p* = 0.003) and the prevalence of obesity was 61% higher (*p* = 0.002), while among females these measures were not significantly different between participants and non-participants. In a study analysing data collected in a survey of low-income neighbourhoods in Massachusetts, which included the US Department of Agriculture (USDA) module on Food Security for Families (HFSM), Web et al. (2008) [19] observed that participation in SNAP or any nutritional assistance program 12 months before the survey was associated with an increase in BMI (3.0 kg/m^2^ more). No association was found between BMI and food insecurity and the individual components of HFSM after adjusting for sociodemographic characteristics and SNAP participation. However, respondents classified as “unsafe” or “hungry insecure” had significantly higher BMI than those classified as “food safe”.

The findings of those studies are consistent with the results of the present study with respect to the association of food assistance public policies with BMI and overweight/obesity, and to the existence of sex differences in that association. However, this question is far from being settled, with conflicting results from several other studies that have shown a greater tendency for overweight and obesity associated with SNAP in women. In a systematic literature review, DeBono (2012) [50] and collaborators suggested that there was evidence of obesity as an unintended consequence of the program, especially for women participating in the long term.

On these different effects of food assistance programs on men and women, some authors [41, 51] suggest that women are significantly more likely to be long-term participants in the program and this period is linked to the increase in BMI. However, this hypothesis does not take into account the response to food insecurity (FI) experienced by women in their families. FI, defined as the lack of consistent access to nutritious foods by socially acceptable ways [52], can, paradoxically, increase BMI by creating a “substitution effect” whereby less expensive and energy dense foods replace healthy foods [53]. A study by Sanjeevi et al. (2018) [54] aimed to examine the role of intrapersonal, domestic, community and social factors in mediating the relationship between FI and diet quality and BMI of 152 low-income women participating in SNAP in Texas central. They noted that the tendency to buy foods high in energy, fat and sugar may be greater in women with FI due to financial constraints. This could explain the greater home availability of unhealthy foods among women with FI observed in this study.

In Brazil, according to Schlüssel et al. (2013) [55], through a study using data from the Brazilian Demographic and Health Survey (DHS), severe FI was associated with a higher prevalence of obesity in adult women (relative risk 1.49). Therefore, our results could be explained by the elimination of FI among females participating in the WFP, which in turn would lead to a lower consumption of macronutrients in this group, compared to women not covered by the WFP, who are more likely to be vulnerable to FI. Thus, our data suggest an interaction between sex and FI that modifies the effect of the WFP on BMI. This hypothesis is supported by a systematic literature review evaluating the evidence basis on the association between FI and weight status. It found that the association was mixed or positively weak among children and men, but was clearly more strongly observed in women, in whom obesity was more likely in those exposed to FI compared to those who have adequate household resources for food [56].

According to Olson [57], women are at greater risk of food insecurity than men, in part due to the responsibility women assume in feeding their families, which makes them responsible, in other words, for managing food insecurity. Indeed, because of the social roles assumed by mothers as providers of their homes, while public food and nutrition security policies are not sufficient or do not produce the expected impact on the food insecurity of the poorest and most vulnerable families, many women will continue to sacrifice your own health for that of your children [57].

Regarding the findings related to food consumption, we observed that male participants in the WFP have greater consumption of carbohydrates and probably also of protein at lunch, the meal offered by the company, than their non-WFP counterparts. Conversely, when compared to the non-WFP group, women of the WFP have lower daily caloric intake and lower consumption of all macronutrients, as well as sodium. The differences in consumption patterns between WFP and non-WFP workers may not be accountable to poor quality of the menus offered, since the caloric and nutritional content of meals and the offer of fruits and vegetables in the menu options is established within the WFP’s Ordinance, which also defines the nutritional monitoring of the population served by the program [10]. We found only one study that included a nutritional evaluation, the previously mentioned study by Chatzivagia (2019) [22], which reported that FEAD participants, compared to non-participants, had lower consumption of total energy and proportionately less proportion of fats and more of carbohydrates and fibre, and that the amount of energy from proteins was higher for FEAD participants, despite a lower protein intake. However, no separate analysis by sex was done.

This study has some limitations to the generalization of the results. The survey was restricted to a single State of the country and the findings found in this region may not replicate in other location, although the results of studies conducted in other States showed results in the same direction of our findings. Only a limited number of sectors of economic activity were surveyed, but this was taken into account in the analysis by including the sector of activity as random factor. The study only assessed refectory meals, whereas the program also includes food baskets and vouchers. In addition, we were careful to consider in the analysis a series of potential confounding factors that could affect the subject’s weight or nutritional status, such as marital status, number of children, education, income, physical activity, among others, but inevitably other unobserved variables can play an important role in this relationship. The use of the 24-hour dietary recall method is known to have limitations related to the interviewee’s memory for identification and quantification of portion sizes [58]. The observational nature of the survey does not allow the establishment of causality between participation in the WFP and differences in nutritional status of workers, but the consistency of such association in studies evaluating the WFP and food assistance programs in other countries gives plausibility to the hypothesis of causality. One issue of cross-sectional studies is the possibility of reverse causality due to preferential participation of workers with a given outcome into one of the study arms. However, this may be excluded in this study because companies, not workers, decide participation in the WFP.

The strong aspects of the methodology include the selection of a probability sample with full coverage of the population of an entire State, the high participation rate, the data collection through direct interview of participants, the design-based analysis of the complex sampling plan, and the dietary assessment by specialized nutritionists with adequate training.

## Conclusion

Among workers assisted by the WFP, a food assistance program in the workplace, compared to workers not assisted by the program, men have higher mean BMI and WC, and greater prevalence of obesity and cardiometabolic risk, while women participating in the program do not have increased BMI and WC, but have lower prevalence of obesity. In the nutritional evaluation, male workers from WFP-participating companies have greater consumption at lunch of protein, carbohydrate and fibre, while women lower daily consumption of energy, carbohydrates, protein and sodium. Our findings suggest that food assistance programs in low-income people achieve their goals mainly through the reduction of food insecurity, rather than to food supplementation to decrease nutritional deficits. If confirmed, this may indicate the need for a change in the selection criteria for participation in food assistance programs where, in addition to low income, the presence of the food insecurity indicator should also be considered, especially among women.

## Supplementary Information

**Additional file 1.**

## Data Availability

All data generated or analysed during this study are included in this published article [and its supplementary information files].

## Abbreviations

WFP: Worker’s Food Program
BMI: Body mass index
WC: waist circumference
NFNP: National Food and Nutrition Policy
FNS: Food and Nutrition Security
HRAF: Human Right to Adequate Food
FIERN: Federation of Industries of the State of Rio Grande do Norte
IPAQ: International Physical Activity Questionnaire
SISVAN: Food Surveillance System and Nutrition
CI: confidence interval
OR: odds ratio
FEAD: Fund for European Aid to the Most Deprived
EU: European Union
SNAP: Supplemental Nutritional Assistance Program
USDA: US Department of Agriculture
FI: food insecurity
